# Carbohydrate-Mediated Modulation of NK Cell Receptor Function: Structural and Functional Influences of Heparan Sulfate Moieties Expressed on NK Cell Surface

**DOI:** 10.3389/fonc.2014.00185

**Published:** 2014-07-16

**Authors:** Michael Brusilovsky, Olga Radinsky, Rami Yossef, Kerry S. Campbell, Angel Porgador

**Affiliations:** ^1^The Shraga Segal Department of Microbiology, Immunology and Genetics, Ben-Gurion University of the Negev, Beer-Sheva, Israel; ^2^The Research Institute of Fox Chase Cancer Center, Philadelphia, PA, USA; ^3^National Institute for Biotechnology in the Negev, Ben-Gurion University of the Negev, Beer-Sheva, Israel

**Keywords:** natural killer cells, natural cytotoxicity receptors, heparan sulfate, heparan sulfate proteoglycans, NKp44, NKp46, NKp30, KIR2DL4

## Heparan Sulfate Glycosaminoglycans

Heparan sulfate (HS) glycosaminoglycans (HSGAGs) are highly complex biopolymers (1, 2). HS structural diversity is characterized by a repeat disaccharide unit of uronic acid (either iduronic or glucuronic acid) linked to a glucosamine (1). Due to the extensive structural diversity of HS resulting from the assembly of 23 distinct disaccharides, it has been called the “most information dense biopolymer in nature” (3, 4), which in turn enables it to interact with a multitude of different proteins. Indeed, the specificity of these interactions would depend on HSGAG composition, tertiary structure, and spacing of binding sites (1).

Heparan sulfate glycosaminoglycans play an essential role in key biological processes and are of particular importance to the survival and progress of various cancers (5). Alterations of the HSGAG epitope repertoire were observed both within various normal tissues and between normal and cancerous tissues (6–8). Indeed, some tumors can exhibit unique carbohydrate profile: over-express certain HSGAGs or express unique HSGAG epitopes (9, 10) that can be rarely found in normal tissue (11). In both tumor and normal mammalian tissue, HSGAGs are usually found covalently attached to various core proteins such as heparan sulfate proteoglycans (HSPGs). The two main groups of cell surface expressed HSPGs are the Syndecans (SDCs) and the Glypicans (1). Bearing in mind the alterations of HSGAG epitope repertoire, HSPGs could be considered as self “modifiable” ligands for HSGAG-binding receptors.

## Natural Killer Cell Receptors and Their Ligands

Natural killer (NK) cells are innate immune cells that are capable of directly attacking tumor, virus-infected, and stressed cells. These functions are controlled by a wide array of germline-encoded activating and inhibitory receptors. NK cell activating receptors include activating forms of killer cell Ig-like receptors (KIR [KIR2DS, KIR3DS, and KIR2DL4]), 2B4, NKG2D, NKp80, and natural cytotoxicity receptor (NCR) -1, -2, and -3 called NKp46, NKp44, and NKp30, respectively. Selective engagement of primary activating receptors such as NCRs can stimulate both cytotoxicity and cytokine production (12–14).

Protein–carbohydrate interactions play a major role in NK cell responses mediated by various activating receptors, including members of the C-type lectins family, such as NKG2D (15–17), and Ig domain-containing family members, like KIR2DL4 and NCRs (12, 18). On the one hand, we and others have shown that NKp46 and NKp44-conjugated glycans are imperative for their interaction with viral hemagglutinins (12). On the other hand, distinct NK cell activating receptors can recognize both proteins (e.g., MICA, HLA-G, B7-H6, and PCNA) and HSGAGs as ligands or co-ligands (2, 15–21). As aforementioned, NCRs were reported to directly bind HSGAGs, yet each of these receptors has been shown to recognize distinct HS structures with fine specificity (2, 22). Both NKp30 and NKp46 recognize highly charged HS/heparin epitopes that are *O*-sulfated at C2 of iduronic acid and bear one to two sulfate groups at the GlcN moiety. However, NKp30 preferentially binds the fully sulfated hexasaccharide, whereas NKp46 interacts more strongly with the analogous tetrasaccharide. In contrast, NKp44 displays a different binding preference toward 2-O-sulfation of IdoA, as well as N-acetylation of GlcN contributing to the binding. Importantly, all the NCRs preferably recognize HSGAGs that are sulfated above average (2).

## Heparan Sulfate Directly Modulates NK Cell Receptor Function

**Figure 1:**
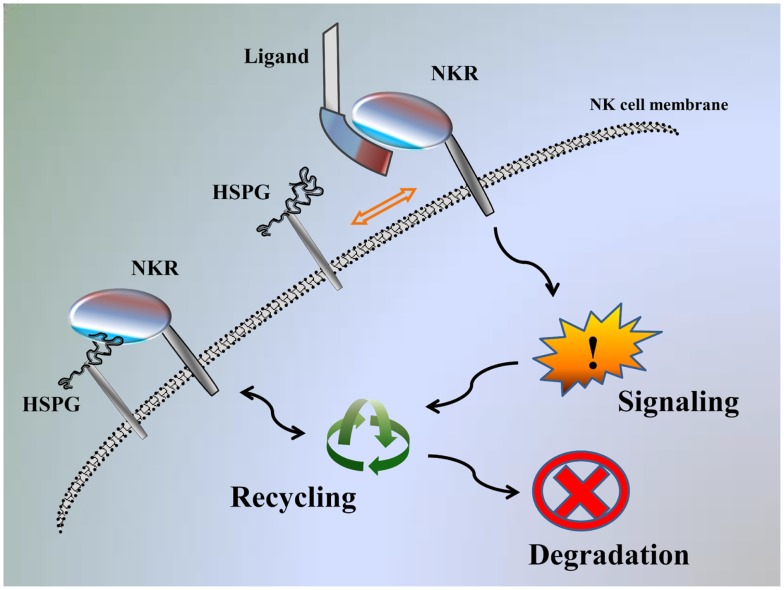
**Model of NK-function autoregulation by HSPG**. Interaction of NKR with the HSPG on the NK cell surface: NKR can be recycled back to the cell surface, promote signaling or destined for degradation. If cells are exposed to ligand stimulation, this displaces HSPG from interacting and NKR is instead available to promote signaling and then can be recycled, or destined for degradation.

We recently demonstrated that KIR2DL4 can also interact with HS/heparin and HSPGs, and these interactions can modulate function of KIR2DL4 to impact NK cell activation, similar to HS-mediated modulation of the function of NCRs (2, 18). The results observed for KIR2DL4 also indicate that NK cell receptor functions may be modulated through interactions with HS either on target cells (-*trans*) or on NK cells themselves (-*cis*). It should also be considered that the high affinity interaction of NCRs/KIR2DL4 with HS/heparin (K_D_ range of 2 μM–20 nM) may, in fact, be adequate to physically engage signaling within an immune synapse upon interaction with HSPG on target cells in trans after exchange from a similar -*cis* interaction with HSPG on the NK cell surface (Figure 1) (18). Therefore, we suggest that HS binding function can both directly engage NK cell receptors to initiate signal transduction and act as an allosteric regulator to modulate the capacity of the receptor to interact with other ligands (18).

Most of the HS on mammalian cells is derived from the SDC HSPGs, and SDC4 in particular (18, 23). We and others have found high expression of SDC4 also in NK cells (18, 24). It has been suggested that SDC4 can oligomerize (23) and may provide a mechanical link between extracellular ligands (i.e., NK receptors (NKR), interacting with HSPG) and the actin cytoskeleton (18, 25–27), and thus stabilize the formation of the receptor–ligand complex as it was previously reported for FGFR (18, 28). Therefore, the primary impact of the NKR–HS interaction could be the autoregulation of the receptor through *-cis* interaction with NK cell-expressed HSPG rather than a trans interaction with target cell-expressed HSPG (18). An analogous mode of *-cis* interaction between NK cell receptor and its ligand could be occurring between Siglec 7 and α2, 8-linked disialic acid structures, while both are widely expressed on the NK cell surface (29).

We have previously reported that exogenous HS can potentiate IFN-γ secretion in NK cells stimulated with specific anti-NKR mAbs (18, 22, 30, 31). We theorize that exogenous HS can block a *-cis* interaction between NKR and HSPG, and therefore releases the receptor, making it available for more efficient engagement by specific mAbs. Here, we theorize that the main function of the NK receptor–HS interaction is to regulate receptor function through the NK cell-expressed HSPG and not through the target cell-expressed HSPG (Figure 1) (18).

Recent advances in clinical research indicate that use of HS and structurally similar Low Molecular Weight Heparin can inhibit tumor progression and metastasis (1). Based upon our data, we postulate that the use of HS/heparin as a therapeutic agent in patients may in fact be significantly altering the activation threshold of the NK cells that express KIR2DL4, NKp44, NKp46, and NKp30. It remains to be determined whether HS/heparin based therapies can regulate these NK cell receptors and modulate NK cell physiology to improve anti-tumor responses. Indeed, the accumulating evidence from our laboratories and others suggests that this possibility should be further explored.

## Conflict of Interest Statement

The authors declare that the research was conducted in the absence of any commercial or financial relationships that could be construed as a potential conflict of interest.
